# Effect of Pretreatments and Solar Tunnel Dryer Zone on Drying Characteristics and Stability of Pumpkin (*Cucurbita maxima*) Slices

**DOI:** 10.1155/2022/5349056

**Published:** 2022-09-16

**Authors:** Hayat Hassen Mohammed, Yetenayet B. Tola, Addisalem Hailu Taye, Zeneba Kedir Abdisa

**Affiliations:** Department of Postharvest Management, College of Agriculture and Veterinary Medicine, Jimma University, Jimma, Ethiopia P.O.BOX 307

## Abstract

Drying fruits and vegetables can be achieved using different drying methods based on the crop's economic value and the technology's affordability. However, in Sub-Saharan Africa, where sunlight intensity and duration are high, it is recommended to use solar drying methods. A solar tunnel dryer is one of the methods commonly used to produce dried fruits and vegetables. It is necessary to determine the drying kinetics at different dryer zones and select a suitable drying kinetics model to overcome the limitation. In addition, pretreatment methods are commonly recommended to improve the quality of the dried product. This work aimed to determine the drying kinetics of pumpkin slices at different zones of drier and pretreatment effect on product quality. Three zones of drier and four pretreatments were employed in the two-factor factorial experiment. Seven thin layer kinetic models were evaluated. pH, TSS, TA, moisture content (MC), and water activity (*a*_w_) were determined for quality evaluation. Recorded data showed that the temperature in the tunnel increased from zone I to III with a decrease in RH. Results showed a higher drying constant (*K*) and effective diffusivity (*D*_eff_); drier zone III > II > I. Pretreatments also showed a significant effect on *K* and *D*_eff_. Regardless of pretreatment types, two-term exponential and diffusion models are better fitted for zones I and II/III, respectively. With pretreatments and drier zones, the TSS value increases from zones I to III but with a decrease in titratable acidity, moisture content, and water activity. From the result, it can be concluded that different drying rates are observed in different zones. However, a better quality of pumpkin powder was observed in drier zone II for pumpkin slices pretreated with a 2% salt solution. It is recommended to create a drying medium that resembles zone II or better to use the recommended kinetic models to predict the drying time for each zone for a better quality product by avoiding under- or over-drying of slices.

## 1. Introduction

Pumpkin (*Cucurbita maxima*) is a fruit vegetable native to South America but has been domesticated in several tropical and subtropical countries [1]. The global production is estimated at more than 27 million tons mainly supplied by China (58%), India (20%), Russia (4%) and Ukraine (4%) [2]. However, no data is available related to Ethiopia production volume even if the fruit produced in different parts of the country. The vegetable is rich in nutrients, adapts well to local conditions, and grows in a wide range of agro-ecological zones [3]. It has tremendous economic potential as a food and cash crop. It is utilized for its leaves, marrow, fruit pulp, and oil seeds [4, 5]. It is rich in minerals, vitamins, pectin, dietary fibers, and vital antioxidants like carotenoids, lutein, and other abundant polyphenolic compounds [6].

It is difficult to store for an extended time because of its large size and its perishable nature [7]. Particularly after peeling, it is susceptible to moisture loss, softening, color change, and microbial spoilage [8]. Moreover, local variety pumpkins, due to their large size (approximately 2-35 kg/fruit), have less consumer acceptance as fresh vegetables [4, 9]; they are difficult for transportation and market [10]. Such problems made pumpkin underutilized vegetables and made it a poor man's food. So far, some attempts have been made to increase the consumption of pumpkins. It includes processing to obtain juice, puree, pickles, and seeds, allowing longer shelf life for the pulp [11]. Despite that, pumpkins having small sizes can be available for fresh markets.

Drying is one of the ancient and vital operations; it involves the application of heat to a material which results in the transfer of moisture within the material to its surface and then water removal from the material to the atmosphere [12–14]. It constitutes an alternative to the consumption of fresh fruits and vegetables and allows their use during the off-production season. Besides giving longer shelf life, it substantially reduces weight and volume, minimizing packaging, storage, and transportation cost and enables safe product storage under ambient temperature [7, 15].

So far, several methods of drying, such as sun drying, convective hot air drying, freeze drying, microwave drying, and vacuum drying, have been studied on the quality of pumpkin [8, 15–17]. To date, solar drying is attracting many scholars in terms of producing relatively better quality dried products as compared to open sun drying, less expensive running costs as compared to convective, freeze, and other advanced drying methods [18, 19].

Among many solar drying methods, a solar tunnel dryer is a commonly used method to dry fruits and vegetables [20]. However, the temperature and relative humidity of the drying medium in long solar tunnel dryers are not uniform. Variations in these factors determine the drying rate and product quality at the end of drying time. For instance, as obtained in the preliminary test, dried samples at the end of the tunnel dryer were exposed to high drying temperature and low relative humidity compared to other drying zones, resulting in variation of the final product quality.

During the drying of food, the food may lose heat-sensitive nutrients, in addition to changes in color and physical properties, depending on the drying condition, such as temperature and drying time [21]. Pretreatment before drying is one of the most critical factors that positively affect the final product quality regarding the physicochemical properties. Pretreatment helps to inhibit spoilage enzymes' negative impact and minimize the degradation of oxygen sensitive vitamins and other health promoting bioactive compounds [22]. Before drying, pretreatments such as blanching and dipping into the appropriate concentration of chemicals (such as citric acid, sodium metabisulfite, and salt) result in minimum quality degradation [23]. Furthermore, pretreatments can extend product shelf life and reduce the drying time of products [17]. Therefore, this work aimed to determine the effect of pretreatment and solar tunnel dryer zones on pumpkins slices' drying characteristics and quality.

## 2. Material and Methods

### 2.1. Description of Experimental Site and Materials

The study was conducted at Jimma University College of Agriculture and Veterinary Medicine (JUCAVM), Food Science, and Postharvest Technology Laboratory. Pumpkins (*Cucurbita maxima*) were collected from the roadside of Woliso, Oromia region, Ethiopia. The selection criteria were based on shape (round), color (light green), and fruit texture. Therefore, fruits having the same shape, color, and texture were collected considering that the selected fruits more or less have a similar genetic makeup and maturity status. The study was conducted at 37° 58′ 23.3832^″^ E, and 8° 32′ 17.9628^″^ N at an elevation of 2039.0 meters above sea level.

### 2.2. Description of Solar Tunnel Dryer

**Figure 1:**
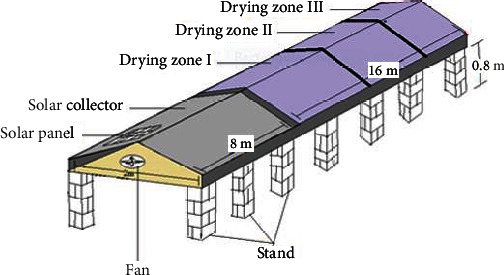
Schematic diagram of solar tunnel dryer.

The solar tunnel dryer has a length of 24 meters and a width of 2 meters, as indicated in Figure 1. It is laid on brick stands, which have a height of 0.8 meters. The solar absorber is 8-m long, and the drying zone is 16-m long. The fan located at the entrance of the solar dryer has a capacity of 75 watts to suck and mix ambient air with air in the absorber section to reduce the very scorching temperature before it enters zone one of the drier. The fan operated by power collected by solar panel (WS 80/85 Mono RHA/D, Germany) is attached at the top of the front side of the absorber. The absorber is black (8-m long and 2-m wide). The total dryer zone of 16 meters is subdivided into three zones, each 5.33-m long, due to variation in temperature and relative humidity obtained in preliminary measurement.

### 2.3. Pretreatments of Pumpkin Pulp

The experiment was performed by selecting three pumpkins of equal size. Then, the selected ones were washed with sufficient tap water. The washed pumpkins were splitted into two halves .Then the rinds, seeds and peels were removed using a knife. Then, pumpkin pulp was uniformly sliced into 2-mm thickness using a vegetable slicer [24]. Then, the slices were treated: in salt solution (2%) for 20 minutes [17]; in 1% citric acid solution for 20 minutes [25]; and blanched in (1%) salt solution at about 65 °C (the temperature was checked by glass thermometer) for 2 minutes [23]. The ratio of 1 : 2 (g of sample/mL of solution) was used in all cases [26].

### 2.4. Drying Process

The drying experiment was conducted between April 16 2020 and April 17 2020, when the condition was fully sunny. After preparing the samples and applying pretreatments, about 0.7 kg of each sample were placed randomly in the three solar tunnel dryer zones. For drying kinetics determination independent representative samples were placed on small wire mesh and weighed using a digital balance (ABJ220-4 M, WB1151070, Australia) with ±0.1 sensitivity at the beginning of drying and in every 30-minute intervals until a constant weight was achieved. During the drying process, the temperature and relative humidity of the solar tunnel dryer were recorded using a data logger (Testo-184H1, Germany). Then after drying stops, the samples were cooled to room temperature overnight, ground into powder, packed in polyethylene bags, and stored at ambient atmospheric conditions during the study period.

### 2.5. Experimental Design

A factorial experiment was laid out considering dryer zones and pre-drying treatments. Solar tunnel dryer levels were three zone I, zone II, and zone III (Table 1). Pre-drying treatment levels were four (untreated (control), 1% citric acid and 2% salt solutions soaked for 20 minutes, and blanched at 65 °C in 1% salt solution). The experiment was laid as a 3 × 4 factorial combination arranged in Randomized Complete Block Design (RCBD) and replicated in 36 experimental units three times.

### 2.6. Collected Data

#### 2.6.1. Drying Characteristics of Pumpkin (*C. maxima*)

*(1) Initial Moisture Content Determination*. The initial moisture content of the fresh pumpkin slice was determined according to AOAC [27] official method number 925.09. Empty dishes were washed and dried using a hot air oven dryer (Leicester, LE675FT, England) at 100°C for 1 h. Then, the dried dishes were cooled in a desicator for 30 min. Each treatment was mixed thoroughly, and 5.00 g of each sample was weighed in triplicate. The dishes and their contents were placed in the drying oven and dried at 10°C to constant weight. After drying, the samples were cooled in desiccators and reweighed until constant weight obtained. Finally, Equation (1) is used to calculate moisture content [28]:

(1) $$\begin{array}{c} MC\left(\%\right)=\frac{M_{i}-M_{d}}{M_{i}}\times 100, \end{array}$$

where *MC* is moisture content, *M*_i_ is the mass of sample before drying, and *M*_d_ is the mass of sample after drying.

*(2) Moisture Ratio*. The moisture ratio (MR) of pumpkin slices during drying experiments are calculated using Equations (2) and (3) according to Goyal et al. [29]:

(2) $$\begin{array}{c} MR=\frac{M_{d}-{\text{M}}_{e}}{M_{o}-M_{e}}, \end{array}$$

where *M*_d_, *M*o, and *M*_e_ are moisture content at any drying time, initial, and equilibrium moisture content (kg water/kg dry matter), respectively. The values of *M*_e_ are relatively small compared to those of *M*_d_ or *M*_o_; the error involved in the simplification is negligible; thus, the moisture ratio is calculated in the following equation [18]:

(3) $$\begin{array}{c} MR=\frac{M_{d}M_{d}}{M_{o}M_{o}}. \end{array}$$

*(3) Effective Moisture Diffusivity (*D*_eff_)*. Fick's second law of diffusion equation, symbolized as a mass-diffusion equation for drying agricultural products in a falling rate period, is shown in the following equation [30]:

(4) $$\begin{array}{c} \frac{\partial M}{\partial t}=D_{eff}\nabla^{2}M. \end{array}$$

The solution of the equation (Equation (4)) for slab geometry is solved by Crank [31] and supposed uniform initial moisture distribution, negligible external resistance, constant diffusivity, and negligible shrinkage:

(5) $$\begin{array}{c} MR=\frac{8}{\pi^{2}}\sum \frac{\partial }{n=0}\frac{1}{{\left(2n+1\right)}^{2}}\exp\left(\frac{{\left(2n+1\right)}^{2}{\pi D}_{eff}t}{4L^{2}}\right), \end{array}$$

where *D*_eff_ is the effective moisture diffusivity (m^2^/s), *t* is the drying time (s), *L* is the half-thickness of samples (m), and *n* is a positive integer.

For long drying times, a limiting of Equation (5) is obtained and expressed in a logarithmic form:

(6) $$\begin{array}{c} \ln\left(MR\right)=\ln\left(\frac{8}{\pi^{2}}\right)-\left(\frac{\pi^{2}D_{eff}t}{4L^{2}}\right). \end{array}$$

From Equation (6), a plot of ln MR versus drying time gave a straight line with a slope (K) of

(7) $$\begin{array}{c} K=\frac{\pi^{2}D_{eff}}{4L^{2}}, \end{array}$$

where *MR* is the moisture ratio, *D*_eff_ is the effective moisture diffusivity (m^2^/s), and *L* is the thickness of the slice of the sample (m).

However, *D*_eff_ of the pumpkin slices was obtained from the slope (*K*) of the ln(MR) graph against the drying time. ln(MR) versus time results in a straight line with a negative slope, and *K* is related to D_*eff*_ by Equation (7) [32].

*(4) Kinetic Model Evaluation*. For drying model selection, drying curves were fitted to seven well-known thin layer drying models, which are given in Table 2. The best-fitted model was determined using four parameters: higher values for the coefficient of determination (*R*^2^) and lower value for reduced Chi-square (*X*^2^), root mean square error (RMSE), and mean relative percent error (*P*) using Equations (7)–(10).

The following thin layer drying models were selected and tested for fitting the models. These were the Lewis, Page, Henderson and Babis, Mindili, logarithmic, and two-term exponential approaches (for more details, see Table 2).

The goodness of fit for each model was evaluated based on the statistical parameters: *R*^2^, RMSE, *χ*^2^, and *P*. The coefficient of determination (*R*^2^) is one of the primary criteria for selecting the best model to define the drying curves. In addition to *R*^2^, reduced Chi-square (*X*^2^), root means square error (RMSE), and relative mean percent error (*P*) are used to determine the quality of the fit [30]. The *R*^2^ value should be higher, and *χ*2, RMSE, and *P* values should be lower. These are calculated in the following equations [33, 34]:

(8) $$\begin{array}{c} R^{2}=1-\frac{\sum_{i=1\;}^{N}{\left({MR}_{\exp,i}-MR_{pre,i}\right)}^{2}}{\sum_{i=1}^{N}{MR}_{\exp,i}-M{R_{\left.pre,i\right)}}^{2}}, \end{array}$$

(9) $$\begin{array}{c} X^{2}=\frac{\sum_{i}^{N}1{\left({MR}_{\exp,i}{MR}_{pre}\right)}^{2}}{N-n}, \end{array}$$

(10) $$\begin{array}{c} RMSE={\left[\frac{1}{N}\overset{N}{\underset{i}{\sum }}=1\;\left({MR}_{pre,i}{MR}_{\exp,i}\right)\right]}^{1/2}, \end{array}$$

(11) $$\begin{array}{c} P\;\left(\%\right)=\frac{100}{N}\sum \frac{\left|{MR}_{\exp}-{MR}_{pre}\right|}{{MR}_{\exp}}, \end{array}$$

where *MR*_exp_ is the ith experimentally observed moisture ratio, *MR*_pre,i_ the ith predicted moisture ratio, *N* is the number of observations, *n* is the number constants, and *X* is the mean [12].

#### 2.6.2. Chemical Properties

*(1) Moisture Content*. Moisture contents of powder pumpkin samples were determined according to AOAC [35], using the official method 925.05. The moisture content is then calculated from Equation (1).

*(2) Total Soluble Solids*. Determination of total soluble solid (TSS) was followed by the method described by Yusufe et al. [36]). First, 2.00 g of pumpkin powder dehydrated to its initial moisture content. Then, the TSS was determined with the help of a hand refractometer (DR201-95, Germany) and was expressed as °Brix (°B) at room temperature. The measurement repeated three times and average values were taken.

*(3) pH and Titratable Acidity (TA)*. The pH value of pumpkin powder was determined following the AOAC [35] official method 981.12 using the pH meter (portable CP-500 Taiwan) which was calibrated with pH 7 and pH 4.

TA was determined according to Pearson and Wong [37] by titrating 50 ml of the homogenate samples against 0.1 N NaOH. First, the distilled water (1 L) used for titration was titrated with 0.1 N NaOH, and the volume of 0.1 N NaOH consumed by water titration was considered a blank. The volume of 0.1 N NaOH used for titration of the sample was noted after correcting the blank, and percent of citric acid is calculated using Equation (12) [38]. In all cases, a triplicate determination was made.

(12) $$\begin{array}{c} Citric\;Acid\left(\%\right)=\frac{V*0.0064*100}{W}, \end{array}$$

where *V* is the volume of 0.1 N NaOH used for sample titration, 0.0064 is the factor equivalent in which 1 ml of 0.1 N NaOH = 0.009008 g C3H6O5, and *W* is the weight in grams of sample in the mixture.

*(4) Water Activity (*a*_w_)*. The water activity was determined by LabMaster-aw instrument (Novasina AG, CH-8853 Lachen, Switzerland) according to Chardelle et al. [39]. The standard cuvette was used in which powder was filled up to the rim and placed below the water activity meter's sensor, which gave direct reading of water activity of the sample.

### 2.7. Statistical Analysis

First, the drying kinetics model (*R*^2^, *X*^2^, *RMSE*, and *P*) values were estimated by nonlinear regression using statistical software (MS, office Excel 2016, and mini tab version 16). Effects of solar tunnel dryer zones and pretreatments on chemical parameters were analyzed using Minitab version 16 whereas ANOVA of a 3 × 4 factorial design for mean separation. The difference between the means was determined using Tukey's test at *α* = 0.05 level of significance.

## 3. Results and Discussion

### 3.1. Variation of Temperature and Relative Humidity in Solar Tunnel Dryer

The temperature and relative humidity of different drier zones, which are the most critical factors for drying kinetics, are presented in Table 1. The highest temperature and the lowest relative humidity were recorded in drier zone III. On other hand, the lowest temperature and highest relative humidity were recorded in ambient air. The air in a solar tunnel drier zone III was 2.2, 1.4, and 1.3 times hotter than ambient, zone I, and zone II, respectively.

### 3.2. Effect of Solar Tunnel Dryer Zones and Pretreatments on Drying Characteristics

#### 3.2.1. Effect of Pretreatments on the Drying Kinetics of Pumpkin Slices

**Figure 2:**
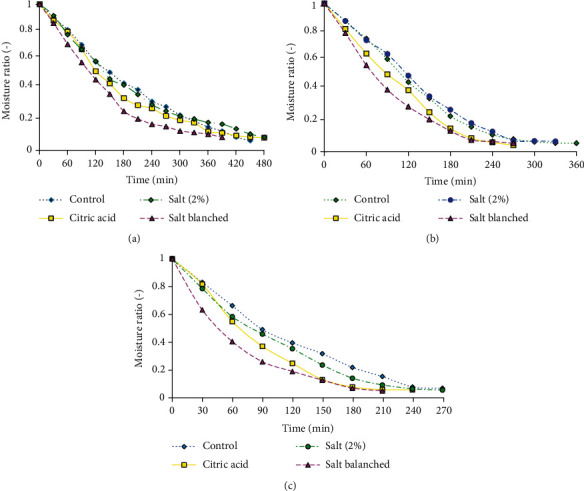
Moisture ratio of pumpkin slices versus time at different pretreatment conditions for zone I of the drier (a), zone II of the drier (b), and zone III of the drier (c). Pretreatments are 1% citric acid solution for 20 minutes, 2% salt solution for 20 minutes, and blanched in 1% salt solution at 65 °C for 2 minutes.

The characteristics of drying curves show the changes in moisture ratio of pretreated pumpkin with time at the three zones of the dryer, as depicted in Figure 2. According to the results, the pretreatment affects the moisture removal of the pumpkin samples as expected. Among the three pretreatments and control, the salt-blanched pumpkin slice shows fast moisture removal in all cases. For instance, in zone I, the salt-blanched sample took the shortest time to reach a moisture ratio of 0.069, and the control sample took relatively more time to dry. The salt-blanched samples experienced higher moisture removal at zone I and zone III. The probable reason that Salt-blanched pumpkin slices dried fast is because hot water containing salt disrupt the cell wall. This improves the movement of water from internal tissue to the surface of the sample. This is supported by Roongruangsri and Bronlund [40], who said, “salt blanching reduces drying time through reducing the firmness of fruit tissue and facilitating moisture diffusion from sample slices.”

The moisture removal at the beginning of drying was fast and became slow as drying time increased. The fast removal of moisture content at the beginning may be due to the surface water on the samples. As the drying process proceeds, the vapor pressure between the samples and air decreases, as the driving force for moisture removal. The result is also confirmed by Kaveh et al. [41] on their investigation of mass transfer, thermodynamics, and greenhouse gasses properties in pennyroyal drying. Generally, the finding of this study was supported by Goyal et al. [29] and Doymaz [30], who observed that various forms of blanching combined with salt pretreatments increase the drying rate for apples and tomato slices.

#### 3.2.2. Effect of Dryer Zones on Drying Kinetics of Pumpkin Slices

**Figure 3:**
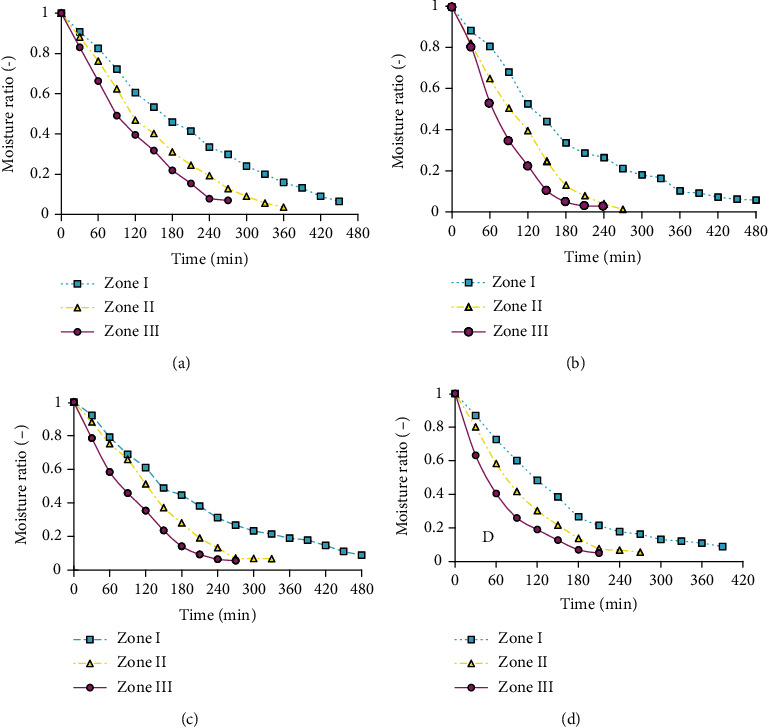
Moisture ratio of pumpkin slices versus time at different dryer zones for control (a), 1% citric acid solution for 20 minutes (b), 2% salt solution for 20 minutes (c), and blanched in 1% salt solution at 65 °C for 2 minutes (d).

Figure 3 depicts the effect of solar tunnel dryer zones on moisture removal of pumpkin slices. As indicated in Figure 3, the pattern of the drying curve for control and citric acid, salt, and salt-blanched-treated pumpkin slices at the three zones was the same. The moisture removal was rapid at zone III and recorded a short time to reach the final moisture ratio. This is because the air in zone III has the highest average temperature and lowest relative humidity relative to the other two zones, which is responsible for the high removal of moisture content from the sample.

As Prachayawarakorn et al. [42] reported, the higher capability of removing moisture at high temperatures due to the acceleration of water molecules produced at higher temperatures took part in a more rapid decrease of the moisture content. This result was also in good agreement with the report of Taheri et al. [43] for tomato slices.

#### 3.2.3. Effective Moisture Diffusivity (*D*_eff_)

Moisture diffusion is considered an essential transport property needed to calculate and model the drying properties of foods and identifies the function of moisture content and temperature in materials [44] The *D*_eff_ of agricultural food product is affected by combination of temperature, relative humidity, and air velocity [18, 45]. The effective moisture diffusivity and drying constant of the pumpkin slices are presented in Table 3. In the present study, the drying constant of the pumpkin slice was found to be between 0.0137 (m^−1^) and 0.0047 (m^−1^) which has a direct effect on effective moisture diffusivity from the slices (Table 4). It revealed that the lowest moisture diffusivity exhibited 7.63 × 10^−9^ (m^2^/s) at zone I for salt (2%) treated slice followed by citric acid treated sample (8.28 × 10^−9^) in the same zone of the dryer. However, the highest moisture diffusivity was 2.34 × 10^−8^ (m^2^/s) for salt blanched followed by a citric acid sample 2.30 × 10^−8^ (m^2^/s) at zone III as indicated in Table 3. This means that high temperature and pre-drying treatment such as blanching in a hot water solution containing salt citric acid facilitates water transport from inside to the pumpkin surface.

The *D*_eff_ values in the present study were in close agreement with values reported by Perez and Schmalko [46] for convective drying of pumpkin as influenced by pretreatment (blanching for one and two minutes) and drying temperature. In addition, Karami et al. [47] observe the increment of *D*_eff_ in the forced convective hybrid-solar dryer during the drying process of rosemary (*Rosmarinus officinalis* l.) leaves. The values of effective diffusivity obtained from this study lie within the general range of 10^−12^ to 10^−8^ (m^2^/s) for drying of food materials [48, 49]. From this result, we can conclude that high temperature and low relative humidity and the application of pre-drying treatments alter effective moisture diffusivity.

#### 3.2.4. Fitting the Proposed Models

Design, simulating, and optimizing the drying process or drying facilities are necessary to obtain drying kinetics data and their modeling. Modeling the drying behavior at the determined condition is essential to obtain higher-quality dried products, which are provided by controlling and optimizing the process parameters [50]. The best model describing the drying characteristics of samples was chosen as the one with the highest coefficient of determination (*R*^2^), the least mean relative percent error (*P*), root mean square error (RMSE), and Chi-square (*X*^2^).

Pumpkin slice dried in zone I was described best by two-term exponential model (Table 5). On the contrary, the diffusion approximation model was found to be the best fitting for describing the drying behavior of pumpkin dried at zone II (relatively medium temperature) (Table 6). Furthermore, considering the mean percent relative error (*P*) value of less than 10, Henderson and Babi's model was suitable for describing the drying characteristics of control and salt-blanched samples. Moreover, a two-term exponential model was found to be suitable for describing the drying behavior of citric acid and salt-blanched samples. Furthermore, the diffusion approximation model best describes the drying behavior of pumpkin slices dried in zones II and III (Tables 6 and 4).

### 3.3. Effect of Solar Tunnel Dryer Zones and Pretreatments on Organoleptic Properties of Pumpkin Powder

#### 3.3.1. Moisture Content and Water Activity

The moisture content and water activity of the dried product are presented in Table 7. The moisture content of the product ranged from 6.4% to 8.2%. The highest value of moisture content and water activity were recorded for the untreated slices dried in a solar tunnel drier zone I. In contrast, the lowest moisture content and water activity were obtained for blanched samples dried in solar tunnel dried zone III. The lowest moisture contents and water activity are directly related to the exposure of the sample to the hottest air having the lowest humidity. The water activity of the dried pumpkin ranged from 0.22 to 0.29, and it is the lowest range for stable storage of pumpkin powder in ambient conditions in recommended packaging material. Water activity has long been considered as one of the most important quality factors for dried products especially for long-term storage with appropriate packaging material [51]. The values of water activity found in the range of 0.20 and 0.40 ensure the stability of the product against browning reactions, lipid oxidation, auto-oxidation, and enzymatic activity [10].

#### 3.3.2. Total Soluble Solid (TSS)

As indicated in Table 7, the results of TSS were found between 7.62 and 11.30. The highest value of TSS was recorded for the citric acid treated samples dried in zone II and zone III and salt treated in zone III. This is related to the fact that one of the functions of salt pretreatment is to improve the TSS of the dried product by preventing the desolation of soluble components. This study followed the result of Teferi et al. [52], who observed the maximum TSS for salt treated and minimum for different untreated accessions of pumpkin powder. The pumpkin powder treated with salt (2%) and citric acid generally showed relatively higher TSS.

#### 3.3.3. pH and Titratable Acidity (TA)

The pH is an important parameter to assess the ability of a microorganism to grow in specific food and decide the food to store under ambient or refrigerated condition [53]. The pH values of dried pumpkins were found in a range of 4.62 to 6.1 (Table 7). The highest value of pH (6.1) and titratable acidity (1.1%) were recorded for the salt pretreated samples and dried in Zone III. Because soaking the samples in salt solution was enough to arrest activity of microorganisms and enzymes that contribute to the conversion of macronutrients into weak acids, weak acid appeared, so the acidity of the product increased.

Drying the samples at highest temperature (zone III) also leads to fast drying and a decrease in conversion of starch into weak acid and as a result decreases in acid production. The decrease in pH for citric acid-treated pumpkin powder is in good agreement with Fana et al. *[*52*]*, who work on orange fleshed sweet potato flour in which citric acid-treated flour shows low pH or high acidity value. This study confirmed that concentration of titratable acidity increases as drying time increases and air temperature decreases. This might be because prolonged drying time causes conversion of macronutrient into weak acids [55].

## 4. Conclusions

Pretreatment and solar tunnel dryer zones were used to investigate their effect on drying characteristics and quality of dried pumpkin slices. Based on study results:

The diffusion approximation model best describes the drying behavior of pumpkin slices at zones II and III. In comparison, a two-term exponential model was suitable to describe the drying behavior of the samples dried in zone I.

Slices blanched in salt solution and dried in zone III experienced high moisture removal rate, short drying time, and high effective moisture diffusivity.

Samples treated with 2% salt solution and dried in zone III resulted in a higher value of TSS and pH. The moisture content and water activity values were recorded for the control sample dried in zone I.

The recorded low water activity values of pumpkin powder were enough to prevent microbial growth and biochemical deterioration.

To maintain uniform drying time and quality, it is recommended to create a drying medium condition resembling zone II or dryer or better to have different drying time at different zones using recommended drying kinetic models to predict the optimum drying time.

## Figures and Tables

**Table 1:** Average, maximum and minimum temperature and relative humidity at three zones and ambient air recorded during drying time.

Zones of solar tunnel dryer	Temperature (°C) and RH (%) in parenthesis^∗^		
	Average	Minimum recorded values	Maximum recorded values
Ambient air	28.27 ± 2.5 (45.73 ± 5.7)	22.9 (36.7)	33 (57.3)
Zone I	45.63 ± 3.38 (34.58 ± 3.67)	40.9 (28.2)	51.6 (42.7)
Zone II	54.78 ± 3.70 (31.36 ± 3.40)	48.9 (25.9)	58.6 (38.6)
Zone III	64.97 ± 6.20 (24.16 ± 9.40)	56 (21.0)	70 (27.0)

^∗^For both temperature and RH, the minimum values were observed in the morning (10 : 00 PM), whereas maximum values were observed from 12 : 00 to 15 : 00 AM. Sample mean expressed ± standard deviation.

**Table 2:** Models and their equation considered for fitting of experiment.

No.	Model name	Model equation	Reference
1	Lewis	MR = exp(−kt)	Doymaz [30]
2	Page	MR = exp(−kt^n^)	Yaldiz and Ertekin [56]
3	Henderson and Babis	MR = aexp(−kt)	Rayaguru and Routray [57]
4	Diffusion approximation	MR = a exp(−kt) + (1 − a) exp(−kbt)	Rayaguru and Routray [57]
5	Mindili et al.	MR = aexp(−kt^n^) + bt	Midilli et al. [58]
6	Logarithmic	MR = a exp(−kt) + c	Kaur and Singh [59]

**Table 3:** Effective moisture diffusivity (D_ef_f) of pumpkin slice at different zones and pretreatments.

Zone	Pretreatment	Drying constant K (-)	*D*_*eff*_(m^2^s^−1^) ± SE × 10^−10^
Zone I	Control	0.0054 ± 0.0003^gh^	8.28 × 10^−9^ ± 4.96^gh^
	1% citric acid	0.0061 ± 0.0003^gf^	9.89 × 10^−9^ ± 4.87^gf^
	2% salt	0.0047 ± 0.0004^h^	7.63 × 10^−9^ ± 5.85^h^
	1% salt blanched	0.0065 ± 0.0003^f^	1.05 × 10^−8^ ± 4.87^f^

Zone II	Control	0.0097 ± 0.0007^ed^	5.7 × 10^−8^ ± 1.14^ed^
	1% citric acid	0.0125 ± 0.0004^b^	2.03 × 10^−8^ ± 6.49^b^
	2% salt	0.0093 ± 0.0005^e^	1.501 × 10^−8^ ± 7.32^e^
	1% salt blanched	0.0115 ± 0.0011^c^	1.87 × 10^−8^ ± 1.84^c^

Zone III			
	Control	0.0102 ± 0.0004^d^	1.65 × 10^−8^ ± 7.07^d^
	1% citric acid	0.0137 ± 0.0002^a^	2.30 × 10^−8^ ± 3.25^a^
	2% salt	0.0114 ± 0.0002^c^	1.85 × 10^−8^ ± 3.25^c^
	1% salt blanched	0.0142 ± 0.0001^a^	2.34 × 10^−8^ ± 1.62^a^

CV (%)		5.104	5.02

LSD		0.0008	67 × 10^−11^

Values expressed are mean values of three replicates ± standard error. All mean scores, bearing different superscript in columns differ significantly (P ≤ 0.05).

**Table 4:** Curve fitting criteria for the various mathematical models and parameters for pumpkin slice dried at zone III.

Pretreatments	Models	Constants	*R* ^2^	*X* ^2^	RMSE	*P* (%)
Control	Logarithmic	a = 1.02136, c = 0.05255, k = 0.0102	0.98994	0.00257	0.04534	21.19
	Henderson and Babi's	a = 1.10852, k =0.0102	0.96619	0.00351	0.05623	13.17
	Lewis	k = 0.0102	0.97312	0.00569	0.07544	17.45
	Page	n = 0.956064, k = 0.0102	0.99157	0.00190	0.04156	21.35
	Two-term exponential	a = 1.61624, k = 0.0102	0.99774	0.00051	0.02144	12.96
	**Diffusion approximation**	a = 1.36744, b = 2.45235 , k = 0.0102	**0.99844**	**0.00039**	**0.01782**	**9.59**
	Mindili et al.	a = 1.3, b = 2.3, n = 0.87, k = 0.0102	0.99083	0.00112	0.02806	7.32

1% citric acid 2% salt	Logarithmic	a = 1.04440, c = 0.04634, k = 0.0137	0.98227	0.00457	0.05856	25.06
	Henderson and Babis	a = 1.10373, k = 0.0137	0.97300	0.00331	0.05425	13.65
	Lewis	k = 0.0137	0.99919	0.00011	0.01062	4.96
	Page	n = 0.923628, k = 0.0137	0.97449	0.00552	0.06946	26.59
	Two-term exponential	a = 1.68565, k = 0.0137	0.99530	0.00103	0.03004	9.45
	**Diffusion approximation**	a = 1.32106, b = 4.78565, k = 0.0137	**0.99920**	**0.00021**	**0.01239**	**7.59**
	Mindili et al.	a = 1.2314, n = 0.97764, b = −0.00019, k = 0.0137	0.96406	0.01088	0.08247	10.33
	Logarithmic	a = 1.02462, c = 0.03513, k = 0.0114	0.99104	0.00198	0.03985	18.03
	Henderson and Babis	a = 1.0828, k = 0.0114	0.97780	0.00238	0.04626	9.93
	Lewis	k = 0.0114	0.98088	0.003498	0.05914	12.56
	Page	n = 0.964683, k = 0.0114	0.99306	0.001367	0.03508	11.45
	Two-term exponential	a = 1.54308, k = 0.0114	0.99750	0.00049	0.02100	8.44
	**Diffusion approximation**	a = 1.31006, b = 2.33796, k = 0.0114	**0.99810**	**0.00042**	**0.01832**	**7.14**
	Mindili et al.	a = 1.06723, b = −0.00026, n = 0.9526, k = 0.0114	0.99585	0.00105	0.02706	11.13

1% salt blanched	Logarithmic	a = 0.990263, c = −0.003414, k = 0.0142	0.99908	0.00017	0.01134	5.01
	Henderson and Babi's	a = 0.9848, k = 0.0142	0.99859	0.00015	0.01157	4.56
	Lewis	k = 0.0142, k = 0.0142	0.99913	0.00011	0.01062	4.96
	Page	n = 1.00640, k = 0.0142	0.99905	0.00015	0.01136	5.14
	Two-term exponential	a = 1.24041, k = 0.0142	0.99901	0.00016	6.123∗10^−5^	4.40
	**Diffusion approximation**	a = 4.16455, b = 0.99344, k = 0.0142	**0.99919**	**6.861**∗**10**^**-5**^	**0.00775**	**3.84**
	Mindili et al.	a = 0.959760, b = 0.000012, n = 0.999209, k = 0.0142	0.99813	0.00042	0.01107	5.07

**Table 5:** Curve fitting criteria for the various mathematical models and parameters for pumpkin slice dried at zone I.

Pretreatments	Models	Constants	*R* ^2^	*X* ^2^	RMSE	*P* (%)
Control	Logarithmic	a = 0.03362, c = 0.0697479, k = 0.0054	0.93780	0.00593	0.07468	17.88
	Henderson and Babi's	a = 1.0299, k = 0.0054	0.98919	0.00287	0.05018	23.43
	Lewis	k = 0.0054	0.96158	0.00918	0.09585	18.8
	Page	n = 0.938821, k = 0.0054	0.99029	0.00242	0.04761	24.86
	**Two-term exponential**	a = 1.7082, k = 0.0054	**0.99834**	**0.00041**	**0.01963**	**9.79**
	Diffusion approximation	a = −15.765, b = 0.990578, k = 0.0054	0.98652	0.00363	0.05639	17.53
	Mindili et al.	a = 1.06982, n = 0.913461, b = −0.000352, k = 0.0054	0.99826	0.00049	0.02014	3.66

1% citric acid	Logarithmic	a = 1.04756, c = −0.01661, k = 0.0061	0.995645	0.00090	0.02910	10.02
	Henderson and Babi's	a = 1.04253, k = 0.0061	0.996707	0.00072	0.02528	9.30
	Lewis	k = 0.0061	0.99549	0.00088	0.02959	11.06
	Page	n = 0.993884, k = 0.0061	0.99527	0.00098	0.03035	7.34
	**Two-term exponential**	a = 1.23268, k = 0.0061	**0.99727**	**0.00067**	**0.02511**	**4.65**
	Diffusion approximation	a = −1.23660, b = 0.99629, k = 0.0061	0.99507	0.00109	0.03102	11.87
	Mindili et al.	a = 1.095, b = 0.00004, n = 1.01556, k = 0.0061	0.99536	0.00109	0.03102	5.10

2% salt	Logarithmic	a = 1.03512, c = −0.00227734, k = 0.0047	0.99628	0.00031	0.01714	5.88
	Henderson and Babi's	a = 1.0299, k = 0.0047	0.99792	0.00047	0.02107	5.32
	Lewis	k = 0.0047	0.99753	0.00053	0.02302	7.23
	Page	n = 0.995076, k = 0.0047	0.99777	0.00051	0.02184	5.10
	**Two-term exponential**	a = 1.0299, k = 0.0047	**0.998557**	**0.00035**	**0.01758**	**4.73**
	Diffusion approximation	a = −2.00076, b = 0.992597, k = 0.0047	0.99785	0.00052	0.02147	4.89
	Mindili et al.	a = 1.07225, b = 0.00006, n = 1.01708, k = 0.0047	0.99786	0.00056	0.00023	4.69

1% salt blanched	Logarithmic	a = 1.05030, c = −0.01453, k = 0.0065	0.99159	0.00078	0.02703	8.60
	Henderson and Babi's	a = 1.02543, k = 0.0065	0.99538	0.00094	0.02949	8.64
	Lewis	k = 0.0065	0.99543	0.00093	0.02953	8.65
	Page	n = 1.00048, k = 0.0065	0.99538	0.00093	0.02954	8.66
	**Two-term exponential**	a = 1.08552, k = 0.0065	**0.99656**	**0.00076**	**0.02560**	**9.27**
	Diffusion approximation	a = −4.22610, b = 0.99913, k = 0.0065	0.99538	0.00102	0.02951	8.65
	Mindili et al.	a = 1.10540, b = 0.00008, n = 1.02952, k = 0.0065	0.99412	0.00140	0.03321	6.14

**Table 6:** Curve fitting criteria for the various mathematical models for pumpkin pretreated and dried at temperatures of zone II.

Pretreatments	Models	Constants	*R* ^2^	*X* ^2^	RMSE	*P* (%)
Control	Logarithmic	a = 1.09905, c = 0.05339, k = 0.0097	0.96643	0.00853	0.08432	19.79
	Henderson and Babis	a = 1.17605, k = 0.0097	0.94332	0.00658	0.07793	5.25
	Lewis	k = 0.0097	0.93227	0.01496	0.12232	22.64
	Page	n = 0.939254, k = 0.0097	0.96672	0.00772	0.08412	15.12
	Two-term exponential	a = 1.86339, k = 0.0097	0.99175	0.00189	0.04163	17.59
	**Diffusion approximation**	a = 1.56798, b = 2.89883, k = 0.0097	**0.99427**	**0.00145**	**0.03475**	**4.56**
	Mindili et al.	a = 1.2202, b = −0.00031, n = 0.944, k = 0.0097	0.97255	0.00768	0.07592	18.86

1% citric acid	Logarithmic	a = 1.05041, c = 0.06769, k = 0.0125	0.96718	0.00825	0.08124	15.70
	Henderson and Babis	a = 1.1674, k = 0.0125	0.91237	0.01031	0.09186	16.98
	Lewis	k = 0.0125	0.88005	0.01532	0.11743	22.37
	Page	n = 0.933899, k = 0.0125	0.97372	0.00588	0.07278	25.58
	Two-term exponential	a = 1.84265, k = 0.0125	0.99339	0.00147	0.03633	9.59
	**Diffusion approximation**	a = 1.69296, b = 2.16407, k = 0.0125	**0.99373**	**0.00157**	**0.03541**	**8.45**
	Mindili et al.	a = 1.1532, b = −0.00050, n = 0.9164, k = 0.0125	0.98170	0.00521	0.06038	24.99

2% salt	Logarithmic	a = 1.07243, c = 0.06214, k = 0.0093	0.97481	0.00672	0.07484	21.56
	Henderson and Babis	a = 1.17788, k = 0.0093	0.93032	0.00796	0.08594	28.09
	Lewis	k = 0.0093	0.94429	0.01287	0.11346	22.34
	Page	n = 0.940650, k = 0.0093	0.97572	0.00590	0.07360	15.99
	Two-term exponential	a = 1.81700, k = 0.0093	0.99479	0.00125	0.03391	8.34
	**Diffusion approximation**	a = 1.54311,b = 2.70822, k = 0.0093	**0.99652**	**0.00093**	**0.02779**	**6.27**
	Mindili et al.	a = 1.15898, b = 0.92535, n = −0.00042, k = 0.0093	0.985904	0.00416	0.05582	11.43

1% salt blanched	Logarithmic	a = 1.04035, c = 0.02073, k = 0.0115	0.99388	0.00132	0.03251	12.35
	Henderson and Babis	a = 1.07466, k = 0.0115	0.98736	0.00139	0.03536	9.11
	Lewis	k = 0.0115	0.98675	0.00196	0.04424	10.56
	Page	n = 0.973267, k = 0.0115	0.99362	0.00122	0.03325	14.49
	Two-term exponential	a = 1.47660, k = 0.0115	0.99721	0.00053	0.02192	8.40
	**Diffusion approximation**	a = 1.17197, b = 5.19267	**0.99940**	**0.00013**	**0.01024**	**6.43**
	Mindili et al.	a = 1.12487, b = −0.00008, n = 0.98773, k = 0.0115	0.99033	0.00237	0.04071	8.59

**Table 7:** Mean of chemical properties and proximate of pumpkin powder obtained from a combination of zone and pretreatments.

Dryer zone	Pre-treatment	TSS (^0^B)	pH	TA (%)	Moisture content (MC) (%)	a_w_
Zone I	Control	7.62 ± 0.006^f^	5.6 ± 0.02^e^	2.09 ± 00.04^d^	8.22 ± 0.03^a^	0.291 ± 0.00^a^
	1% citric acid	10.20 ± 0.03^b^	4.62 ± 0.01^h^	2.68 ± 0.02^a^	6.89 ± 0.02^g^	0.29 ± 0.0007^a^
	2% salt	9.22 + 0.08^c^	5.9 ± 0.05^bc^	1.8 ± 0.02^ef^	7.24 ± 0.05^de^	0.274 ± 0.0008^cd^
	1% salt blanch	7.69 ± 0.04^f^	5.3 ± 0.03^f^	2.1 ± 0.04^d^	6.65 ± 0.02^h^	0.282 ± 0.001^b^

Zone II	Control	10.14 ± 0.03^b^	5.7 ± 0.04^de^	1.40 ± 0.008^h^	7.75 ± 0.02^b^	0.263 ± 0.0006^ef^
	1% citric acid	11.2 ± 0.02^a^	4.8 ± 0.02^g^	2.50 ± 0.003^b^	7.40 ± 0.06^cd^	0.267 ± 0.002^cde^
	2% salt	10.24 ± 0.04^b^	6.0 ± 0.01^ab^	1.3 ± 0.03^h^	7.14 ± 0.08^ef^	0.274 ± 0.0008^cd^
	1% salt blanch	8.21 ± 0.02^e^	5.39 ± 0.009^f^	1.7 ± 0.05^fg^	7.57 ± 0.02^bc^	0.266 ± 0.002^de^

Zone III	Control	10.12 ± 0.08^b^	5.8 ± 0.01^cd^	1.6 ± 0.05^g^	7.00 ± 0.04^fg^	0.241 ± 0.002^g^
	1% citric acid	11.3 ± 0.03^a^	4.9 ± 0.02^g^	2.50 ± 0.003^b^	6.76 ± 0.04^gh^	0.255 ± 0.001^f^
	2% salt	11.13 ± 0.06^a^	6.1 ± 0.03^a^	1.1 ± 0.04^i^	6.96 ± 0.04^fg^	0.257 ± 0.003^f^
	1% salt blanch	8.92 ± 0.03^d^	5.8 ± 0.02^cd^	1.9 ± 0.02^e^	6.38 ± 0.04^i^	0.227 ± 0.002^h^

	CV (%)	0.63	0.74	2.79	1.02	0.92

Values expressed are mean values of three replicates ± standard deviation. All mean scores, bearing different superscript in columns differ significantly (P ≤ 0.05).

## Data Availability

The datasets supporting the conclusions of this article are included within the article and could be available from the corresponding author upon request.
